# Validation Studies for Single Circulating Trophoblast Genetic Testing as a Form of Noninvasive Prenatal Diagnosis

**DOI:** 10.1016/j.ajhg.2019.11.004

**Published:** 2019-11-27

**Authors:** Liesbeth Vossaert, Qun Wang, Roseen Salman, Anne K. McCombs, Vipulkumar Patel, Chunjing Qu, Michael A. Mancini, Dean P. Edwards, Anna Malovannaya, Pengfei Liu, Chad A. Shaw, Brynn Levy, Ronald J. Wapner, Weimin Bi, Amy M. Breman, Ignatia B. Van den Veyver, Arthur L. Beaudet

**Affiliations:** 1 Department of Molecular and Human Genetics, Baylor College of Medicine, Houston, TX 77030, USA; 2 Baylor Genetics, Houston, TX 77021, USA; 3 Department of Molecular and Cellular Biology, Baylor College of Medicine, Houston, TX 77030, USA; 4 Department of Biochemistry, Baylor College of Medicine, Houston, TX 77030, USA; 5 Department of Pathology and Cell Biology, Columbia University Medical Center, New York City, NY 10032, USA; 6 Department of Obstetrics and Gynecology, Columbia University Medical Center, New York City, NY 10032, USA; 7 Department of Medical and Molecular Genetics, Indiana University School of Medicine, Indianapolis, IN 46202, USA; 8 Department of Obstetrics and Gynecology, Baylor College of Medicine, Houston, TX 77030, USA; 9 Texas Children’s Hospital, Houston, TX 77030, USA

**Keywords:** noninvasive prenatal diagnosis, cell-based NIPT, fetal, single cell analysis, trophoblast, mosaisicm

## Abstract

It has long been appreciated that genetic analysis of fetal or trophoblast cells in maternal blood could revolutionize prenatal diagnosis. We implemented a protocol for single circulating trophoblast (SCT) testing using positive selection by magnetic-activated cell sorting and single-cell low-coverage whole-genome sequencing to detect fetal aneuploidies and copy-number variants (CNVs) at ∼1 Mb resolution. In 95 validation cases, we identified on average 0.20 putative trophoblasts/mL, of which 55% were of high quality and scorable for both aneuploidy and CNVs. We emphasize the importance of analyzing individual cells because some cells are apoptotic, in S-phase, or otherwise of poor quality. When two or more high-quality trophoblast cells were available for singleton pregnancies, there was complete concordance between all trophoblasts unless there was evidence of confined placental mosaicism. SCT results were highly concordant with available clinical data from chorionic villus sampling (CVS) or amniocentesis procedures. Although determining the exact sensitivity and specificity will require more data, this study further supports the potential for SCT testing to become a diagnostic prenatal test.

## Introduction

Circulating cells from the fetus or placenta in a pregnant woman’s blood—primarily trophoblasts and nucleated fetal red blood cells (fnRBCs)—have long been appreciated as potential targets for noninvasive prenatal testing (NIPT) and diagnosis. Upon isolation, these cells provide a pure source of fetal genomic DNA, whereas in the current fetal cell-free DNA (cfDNA)-based test (“cell-free NIPT”) the fetal fraction only contributes 5% to 20% of the total cfDNA, thus hindering the resolution, specificity, and utility of the test. In addition, several factors, such as maternal malignancies, maternal chromosomal mosaicism, maternal copy-number variants (CNVs), maternal body mass index (BMI), and maternal organ transplants, can substantially affect the data. This can compromise the test result, leading to false-positive aneuploidy or CNV results and, more rarely, false-negative results.^1^, ^2^, ^3^, ^4^ Hence, cell-free NIPT is considered a screening test requiring diagnostic confirmation, whereas cell-based NIPT has the potential to become a diagnostic test.

The primary challenge for cell-based NIPT is that the target cells are exceedingly rare at 1–2 cells/mL maternal blood,^5^ and there is considerable inter-individual variability in the number of recoverable cells. However, despite the fact that the relative scarcity of these cells makes their isolation process challenging, we and others have previously published evidence for the feasibility of trophoblast-based NIPT for fetal CNV diagnosis.^6^, ^7^, ^8^, ^9^

Although much of our work has utilized a maternal-white-blood-cell-depletion method to enrich for target-cell candidates,^6^^,^^9^ we have also collaborated on a positive enrichment strategy using magnetic-activated cell sorting (MACS).^7^ In this report, we have returned to positive selection with MACS. We found that the positive selection method was simpler and recovered slightly greater numbers of trophoblasts. Here, we have performed the largest study to date and two validation studies in which this positive trophoblast selection was applied serially to a set of maternal venous blood samples that were subsequently analyzed by whole-genome sequencing for genome-wide CNV analysis. Given the potential variation in the quality of singlecells,^9^ we opted to analyze each cell individually, unless they were present in a cell cluster that did not dissociate during the cell-picking process. Clusters include doublets where the two cells often appear as pairs of mirror images, possibly being two daughter cells from a recent cell division. Some higher-order clusters were seen, and we assume that these cell clusters came free of the placenta as a cluster as opposed to having been formed as aggregates while in the circulation. This also allows for adequate genotyping to confirm the fetal origin of each cell and to potentially distinguish different genetic signatures in twin or higher-multiple pregnancies or in the case of confined placental mosaicism. Hence, we refer to our assay as single circulating trophoblast (SCT) testing hereafter.

## Material and Methods

### Sample Collection

After informed consent was obtained, blood samples from pregnant women were collected in accordance with the ethical standards of the responsible committee on human experimentation under a protocol approved by the institutional review boards of Baylor College of Medicine and Columbia University. Virtually any woman presenting for prenatal care was eligible for inclusion, although there was some preference for women in their first-trimester. Women were excluded only if blood draw was not possible. Most of the women were recruited in Houston when being seen in clinic for genetic counseling and decision making regarding prenatal testing. In New York, pregnant women were recruited at the time of an appointment for CVS or amniocentesis. The range of gestational age is given in Table 1 and listed specifically for every sample in Table S1. For study 1, maternal blood samples were collected in three 10 mL EDTA vacutainer tubes (for trophoblast cell enrichment) and one 4 mL tube (for extraction of maternal genomic [g] DNA and fetal *cf.*DNA for fetal sex determination). For study 2, the volume was increased to four 10 mL tubes and one 4 mL tube,. Whenever available, paternal samples (saliva or 2 mL EDTA) were collected. Samples collected at the Columbia recruitment site were shipped overnight to Houston at ambient temperature to be processed the next day. Tables 1 and S1 give both a summarized and detailed overview, respectively, of all sample information for both studies. Control samples from healthy, non-pregnant individuals were collected for lymphoblast spike-in experiments.

**Table 1:** Overview of Sample Characteristics for Both Studies

**Parameter**	**Study 1**	**Study 2**
Number of samples	42	53
Average sample volume	28.5 mL, ± 3.1 mL	36.2 mL, ± 4.3 mL
Gestational age	8 weeks, 2 days to 20 weeks, 6 days (average: 13 weeks, 2 days, ± 2 weeks, 5 days)	9 weeks, 3 days to 29 weeks, 5 days (average: 13 weeks, 6 days, ± 3 weeks, 5 days)
Plurality and Fetal sex	22 F singletons/18 M singletons/2 twins (1 F+M and 1 M+M)	20 F singletons/27 M singletons/6 twins (4 F+M, 1 M+M, and 1 F+F)
Recruitment site	29 Houston; 13 New York	39 Houston; 14 New York
Maternal age	26–43 years old (average: 34.7 years old, ± 3.9 years)	19–41 years old (average: 33.1 years old, ± 5.2 years)
Maternal BMI	19.0–42.7 kg/m^2^ (average: 26.3 kg/m^2^, ± 5.4 kg/m^2^)	18.9–41.3 kg/m^2^ (average: 27.3 kg/m^2^, ± 6.0 kg/m^2^)
Clinical diagnostic testing	19 procedures: 13 CVS (of which 6 blood samples were collected post-CVS); 5 amniocentesis (none were collected post-amnio); 1 both (sample was collected after amniocentesis procedure) // 23 no procedure	25 procedures: 14 CVS (of which 9 samples were collected post-CVS); 10 amniocentesis (of which 3 samples were collected post-amnio); 1 both (sample was collected after CVS procedure) // 28 no procedure

**Main Indication for Genetic Counseling and/or Diagnostic Testing**		

AMA	23 (54.8%)	20 (37.7%)
Abnormal cell-free NIPT	3 (7.1%)	3 (5.7%)
Abnormal ultrasound	3 (7.1%)	9 (17.0%)
Parental indication (balanced translocation, carrier)	3 (7.1%)	3 (5.7%)
Regular first trimester screen (not AMA)	3 (7.1%)	7 (13.2%)
Abnormal prior pregnancy	1 (2.4%)	3 (5.7%)
Abnormal quad screen	1 (2.4%)	-
Abnormal CVS	1 (2.4%)	-
Abnormal serum screen	-	1 (1.9%)
Abnormal PGT result	-	1 (1.9%)
Combination of 2 or 3 of the above	4 (9.5%)	6 (11.3%)

**Trophoblast Yield as Identified by Microscopy**		

Total	226 cells	398 cells
Per sample: average	5.38 cells/sample	7.51 cells/sample
Per sample: median	3.00 cells/sample	6.00 cells/sample
Per sample: range	1–23 cells/sample	0–26 cells/sample
Per mL maternal blood: average	0.19 cells/mL	0.21 cells/mL
Per mL maternal blood: median	0.11 cells/mL	0.18 cells/mL
Number of samples with cell clusters	18 (43%)	28 (53%)

^∗^AMA, advanced maternal age; BMI, body mass index; CVS, chorionic villi sampling; F, female; M, male; NIPT, noninvasive prenatal testing; PGT, preimplantation genetic testing; SD, standard deviation

### Genomic DNA Extraction and Fetal Sex Determination

Maternal and, when available, paternal gDNA was extracted from whole blood on a MagnaPure platform (Roche) via a MagnaPure compact nucleic acid isolation kit I. If saliva from the father was available, gDNA was extracted with a MagnaPure compact nucleic acid isolation kit I, large volume, on the same platform.

Fetal cfDNA was extracted from maternal plasma on the aforementioned platform as well, also via the large volume kit. This cfDNA was used in a Y chromosome qPCR reaction so that the fetal sex could be determined on the basis of the detection two amplicons for *DYS14* and *SRY*, as has been described.^6^^,^^9^

### Trophoblast Enrichment

Blood processing was started at most 24 h after sample collection, except for one sample for which transport was delayed and processing started 2 days after collection. In a first step, the blood sample underwent a 10-min fixation by the addition of a 0.67× volume of 5% paraformaldehyde in PBS. Subsequently, the red blood cells were lysed by incubating for 8 min with 10× the original blood volume of red blood cell (RBC) lysis buffer containing 0.12% Triton X-100 in PBS. These two initial incubations took place at room temperature on a tube roller (20 rolls/min). Then 5× original blood volume of PBS containing 2% BSA was added. The sample was centrifuged (700 × g, 15 min, 4°C) in two 500 mL bottles, and the supernatant was aspirated down to a final volume of 5 mL per bottle. The remaining cell pellets were combined, underwent two washing steps with PBS, and were reduced to 1 mL in PBS; then a 3 mL MACS separation buffer (Miltenyi Biotec) was added.

A cocktail containing 4 μg each of four biotinylated enrichment antibodies was added to each sample and incubated for two h at 4°C on a laboratory shaker. The three commercial enrichment antibodies were biotinylated mouse anti-human HLA-G, biotinylated mouse anti-human TROP-2 (both Novus Biologicals), and biotinylated mouse anti-human EpCAM (BioLegend). The fourth antibody was developed against JEG cell line membranes by the Protein and Monoclonal Antibody Production Core at Baylor College of Medicine. Screening of this antibody was performed by the Mancini lab and the Antibody core facility, and subsequent mass spectroscopy analysis was used for identification of its antigen, which was EpCAM. The fourth antibody (up to 2 mg) was biotinylated via the Lightning-Link Rapid Biotin Kit type A from Expedeon. Subsequently, samples were incubated with anti-biotin magnetic microbeads (Miltenyi Biotec) for two h at 4°C on a laboratory shaker. Positive trophoblast selection was achieved with MS columns and a MiniMACS separator (Miltenyi Biotech). After enrichment, the resulting cells were resuspended in BD Perm/Wash buffer (BD Biosciences) and underwent immunostaining with two anti-cytokeratin antibodies (both pan-CK reactive, BioLegend and eBioScience), one antibody against the white blood cell (WBC) marker CD45 (BioLegend), and DAPI nuclear stain (1-h incubation at room temperature).

### Trophoblast Isolation and Single-Cell Processing

After immunostaining, the sample was spread into CyteSlides (RareCyte) in PBS, and these were subsequently scanned on a CyteFinder (RareCyte). All identified trophoblast candidates were then curated manually on the basis of specific criteria (positive cytokeratin staining and its pattern, negative for CD45, nuclear morphology) as described previously.^6^^,^^9^ Using the CytePicker module (RareCyte), all putative trophoblasts were picked individually with a 40-μm diameter needle and deposited into PCR tubes in 2 μL PBS. All cells were stored at −80°C until further processing.

Before downstream analysis, each cell underwent whole-genome amplification (WGA) via the PicoPLEX WGA kit (Takara Bio). The concentration of the amplified DNA was measured on a Nanodrop platform.

### Cell Culture for Spike-in Experiments

All lymphoblast cell lines were kept in culture under normoxic conditions (37**°**C, 5% CO_2_) in fetal bovine serum (FBS)-supplemented RPMI 1640 culture medium. Three different lymphoblast cell lines were included in spike-in experiments: a male line harboring a 3.8 Mb Smith-Magenis syndrome deletion (17p11.2) (MIM: 182290), a female line harboring a 2.5 Mb DiGeorge syndrome deletion (22q11.21) (MIM: 188400), and a female line harboring a 1.3 Mb Charcot-Marie-Tooth duplication (17p12) (MIM: 118220). Before being spiked into control blood, an aliquot of each cell culture was labeled with both CellTracker Green 5-chloromethylfluorescein diacetate (CMFDA; Life Technologies) and MitoTracker Orange chloromethyltetramethylrosamine (CMTMRos; Life Technologies) according to the manufacturer’s instructions so that the spiked-in cells in the eventual enriched cell suspension could be easily detected via microscopy. Single lymphoblasts were picked in the same manner as trophoblasts and stored at −80°C until WGA processing and further analysis.

### Next-Generation Sequencing and CNV Analysis

All next-generation sequencing (NGS) was performed on WGA products from single cells, or occasionally from cell clusters. No single cells or WGA products from singleton or twin pregnancies were pooled. After consecutive DNA shearing (Covaris), end repair (New England Biolabs reagents), A-tailing (NEB), and Illumina adaptor ligation, a round of PCR with specific Illumina primers was performed. Once the library preparation was finished, single-end sequencing (read length 100 bp) was performed for all samples on a HiSeq 2500 platform (Illumina); the desired coverage was 5 × 10^6^ reads/sample. Sequence files were mapped against the human reference genome (hg19) via the Burrows-Wheeler Aligner MEM algorithm (v.0.7.15). Coverage counts were generated with the bedtools’ (v.2.25.0) multicov function.

All generated. bam files were analyzed in NxClinical (BioDiscovery). Each trophoblast was compared to a 3-cell pool of normal female trophoblast reference and to a 3-cell pool of normal male trophoblast reference for genome-wide CNV analysis. This software enables multiple modes of data visualization, including whole-genome plots that allow for nucleotide-level zooming in on the data for each cell and multi-sample views indicating the copy-number changes automatically called within the software.

Two American Board of Medical Genetics and Genomics (ABMGG)-certified directors (A.M.B. cytogenetic and W.B. combined cytogenetics and molecular genetics) reviewed all data in a blinded manner to score each cell for its quality and judged whether they were scorable for both aneuploidy and smaller CNVs (≥1 Mb) detection on the basis of their NxClinical quality score and whole-genome profile.

### Genotyping

Application of an in-house-developed genotyping assay confirmed the fetal origin of the analyzed genome.^9^ A multiplex PCR including 41 amplicons was run on the WGA products and maternal gDNA (as well as paternal gDNA when available), and the resulting products underwent MiSeq sequencing (2 × 150 bp paired-end reads). The SNP profiles were subsequently compared with the maternal profile. In case a fetal allele for a given SNP is different from the maternal profile, this demonstrates the fetal origin of the cell.

## Results

### Trophoblast Identification and Yield

An overview of all sample information for both validation studies is summarized in Table 1, and complete data are provided in Table S1. For study 1, a total of 226 trophoblasts were microscopically identified in 42 samples with 5.38 cells/sample on average (5.08 cells/sample for singletons only). Recalculated as cells per mL of maternal blood (an average volume of 28.5 mL per sample was processed), this is 0.19 cells/mL on average (0.18 for singletons). Cells were found in all samples included in this study set, and the range of trophoblasts identified per sample was 1 to 23 cells. In study 2, the blood volume was increased to four 10 mL EDTA tubes; there was an average of 36.2 mL per sample, and 398 trophoblasts were identified in total in 53 samples. This number correlates to 7.51 cells/sample on average (6.91 for singletons) and to an average of 0.21 cells/mL (0.18 for singletons). The range was 0 to 26 cells, and this set included two samples for which no cells were found.

**Figure 1:**
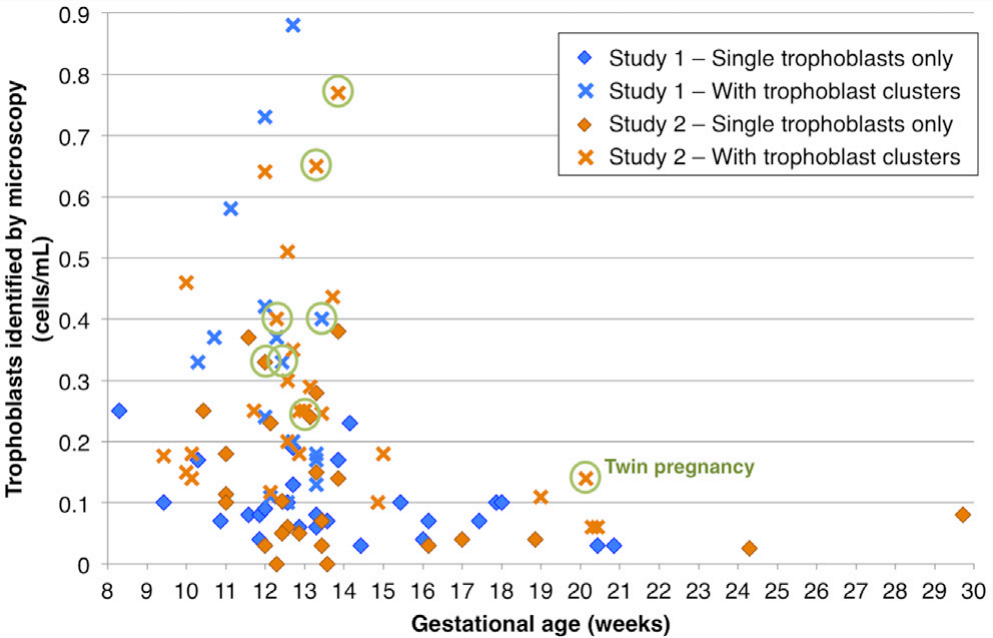
Number of Trophoblasts Identified by Microscopy The total number of putative trophoblasts/mL of maternal blood is shown with coding according to the study and gestational age of each sample. “Clusters” means that groups of two or more trophoblasts were detected and processed as a unit if they did not separate during picking as described in the Material and Methods. About 55% of these cells will give NGS data that are scorable for both aneuploidy and pathogenic CNVs. All twin pregnancies are indicated with a circle.

Figure 1 displays the number of trophoblasts microscopically identified per sample for both studies and also indicates whether only single cells or also trophoblast clusters were found. Trophoblast identification relied primarily on cytokeratin (CK) positivity and CD45 negativity. For study 1, clusters were found in 18/42 samples (43%); in study 2 clusters were found in 28/53 (53%) (Table 1). The two singleton pregnancies for whom no cells were recovered in study 2 were NIPT1484, age 37 at 13 weeks 4 days gestational age (GA) and having a BMI of 30.95 kg/m^2^, and NIPT1497, age 39 at 12 weeks, 2 days GA and having a BMI of 38.87 kg/m^2^. Neither of them was associated with any unusual circumstances, and we suspect that the number of cells recovered might be based on normal biological variation in placentation. In other studies in our lab, fewer cells were recovered between 7 weeks, 0 days and 10 weeks, 0 days of gestation (data not shown) and after 15 weeks of gestation. We found a statistically significant downward trend in trophoblast cells identified with increasing GA for these datasets: when we considered all samples from both studies together, the correlation coefficient for cells/mL as a function of gestation was −0.36, with a p value of 0.00032 (Spearman’s correlation, 95 singleton and twin samples in total; r = −0.41 for singletons only, p value 7 × 10^−5^). When we divided the singleton data into two groups, one group including samples collected at ≤14 weeks and 6 days (70 samples) and one group at ≥15 weeks (17 samples), the mean for the samples collected at earlier gestation (0.21 cells/mL) was significantly higher than for those collected at later gestation (0.07 cells/mL; p value = 0.00006, Mann-Whitney test). There were considerably fewer samples collected at a later gestation. We presume that the fewer cells recovered at later gestations reflects normal placental development. We conclude that SCT testing is optimally used at 15 weeks of gestation or earlier. If used at later gestations, physicians and patients should be aware of a lower success rate.

Maternal BMI data were available for 85 out of the 95 samples; the average maternal BMI was 26.8 kg/m^2^ (range = 18.9–42.7). Although there was a downward trend for cell recovery with increasing BMI, the difference was not statistically significant (Spearman’s r = −0.203, p = value 0.063; r = −0.206 for singletons only, p value = 0.071). No correlation was found with maternal age (Spearman’s r = −0.05, p value = 0.657; all singleton and twin pregnancies were included).

In total, 44 women underwent CVS or amniocentesis. Although blood draw prior to procedure was recommended, for 20 of those, the blood sample could only be collected shortly after the procedure (within minutes to up to two h). However, there was no statistically significant difference in trophoblast yield when blood samples collected after CVS or amniocentesis were compared to all other samples (Mann-Whitney; p value 0.960). Resources did not permit following up on all infants at birth. Therefore, conclusions about accuracy of SCT testing were made only on the basis of data from the pregnancies where CVS or amniocentesis was also performed. There were two samples (NIPT1310 and NIPT1312) where the SCT test result showed trisomy 21 and the cell-free NIPT result indicated trisomy 21, and both pregnancies were terminated on the basis of cell-free NIPT and ultrasound findings without CVS, amniocentesis, or product of conception testing. These were not included in calculating accuracy.

### Confirmation of Fetal-Cell Origin

For the total of 149 putative trophoblasts analyzed from confirmed male singleton pregnancies, nine cells were found to be of maternal origin by the presence of a 46,XX complement on their next-generation sequencing (NGS) profile. Thus, 94% of cells scored microscopically as fetal were male. For pregnancies with a female fetus, all cells were genotyped so that maternal origin could be distinguished from fetal origin as previously described.^6^^,^^9^ For 155 cells that were judged to be female on the basis of their CNV analysis result and that came from pregnancies with a female fetus or twins, genotyping confirmed that 149 (96%) were of fetal origin.

### Twin Pregnancies

In total, eight twin pregnancies were analyzed, as detailed in Table S2: five dichorionic-diamniotic opposite-sex twin pairs, two male-male pairs (one dichorionic-diamniotic and one monochorionic-diamniotic), and one dichorionic-diamniotic female-female twin pregnancy. Not unexpectedly, we found a higher cell number for these twin pregnancies: the average for all eight twin pregnancies was 13.5 cells/sample and 0.41 cells/mL (versus 0.18 for singletons). The range of trophoblasts microscopically identified was 3–26 cells/sample, or 0.11–0.77 cells/mL. When comparing these values to the group of 87 singleton samples, we found the difference to be significant with a p value of 0.001 (Mann-Whitney test).

**Figure 2:**
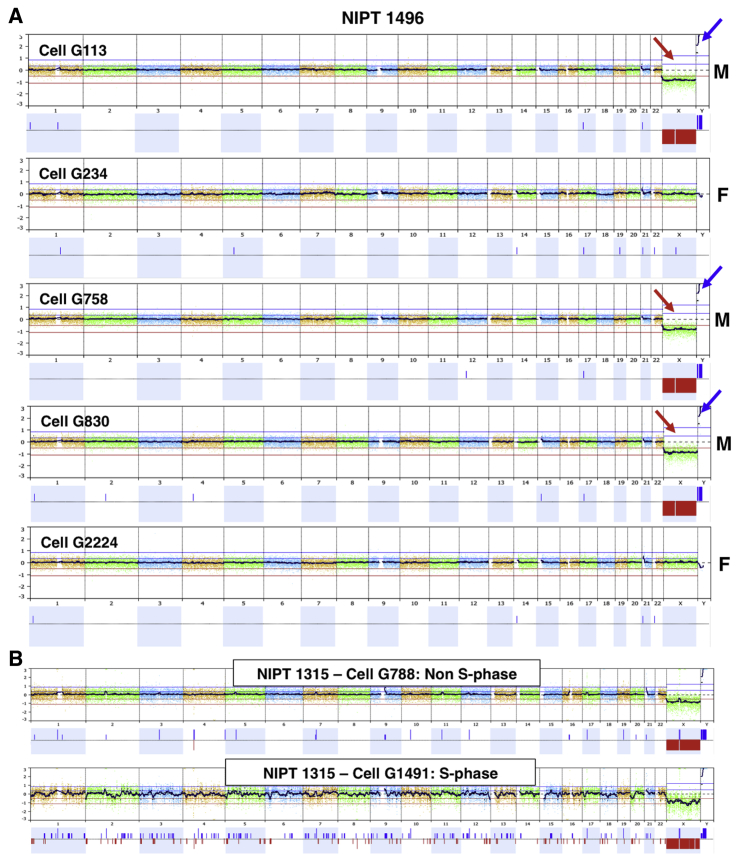
Single-Cell NGS Analysis and Single-Cell Quality (A) Multiple cells from an opposite-sex twin pair are demonstrated. Each cell is compared to a normal female control, and a loss of X (red arrows) and gain in Y (blue arrows) is seen for three male cells. All cells shown were judged to be scorable for both aneuploidy and pathogenic CNVs. All cells were genotyped as fetal, and the two female cells are from the other twin. The clinical result for both twins was interpreted as normal. (B) An example of a single S-phase cell compared to a cell not in S phase. The fetus is male, and both cells are compared to a normal female control. The upper cell is judged to be scorable for both aneuploidy and pathogenic CNVs, whereas the lower cell is judged to be in S phase and scorable for aneuploidy but not for CNVs.

For NGS analysis on twins, a minimum of five cells per sample was included, except for one sample (NIPT1432, female-male twin pair) for which only one female doublet was available for downstream analysis. For three other opposite-sex twin pairs, cells from both the male and female fetuses were recovered, whereas for the fifth twin pregnancy (NIPT1441) 10 cells were analyzed, and all were female. Cells from both sexes from one of the opposite-sex twin pairs are illustrated in Figure 2A.

### NGS Analysis and Single-Cell Quality

Single-cell sequencing data can vary considerably in terms of quality. We found some cells to be in a state indicative of apoptosis on the basis of their sequencing profile. As reported previously,^9^ apoptotic cells have widely variable copy number; often entire chromosomes vary such that there are chromosomes with 2, 1, and even 0 copy number. This is consistent with published evidence that chromosomes are often degraded as entire domains in apoptotic cells.^10^ For study 1, this was the case for 9.3% of analyzed cells; for study 2, this was the case for 6.67% of cells. Additionally, the profile for a portion of cells suggested that these were in the S phase of the cell cycle. We interpreted cells with a bimodal copy number across the entire genome as indicative of S phase, which is illustrated in Figure 2B (cell G1491). For cells in late S phase, yet-to-be-replicated regions were indistinguishable from what might be small deletions across the entire genome. We saw an indication of S phase for 11.0% of the cells analyzed in study 1, and for 17.1% of the cells in study 2. Upon analysis, we found that these S-phase cells are still adequate for aneuploidy scoring in more than 90% of cells (Figure S1).

**Figure 3:**
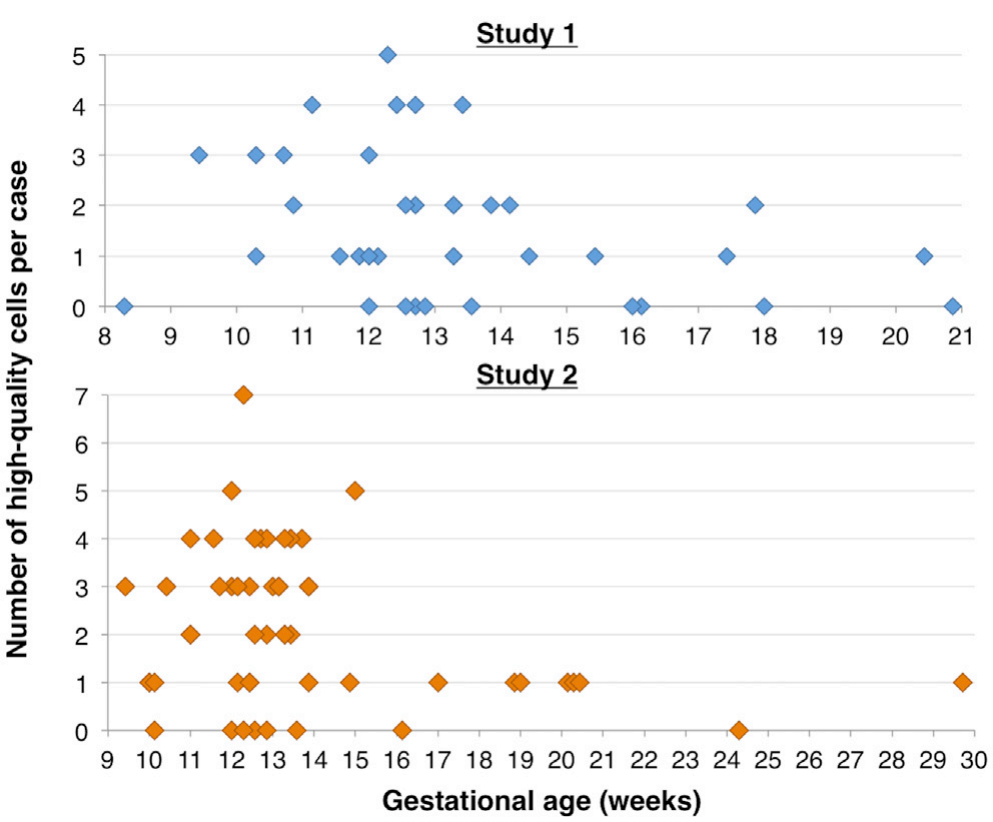
Yield of High-Quality Cells for Both Studies These two graphs show the number of high-quality cells, i.e., two blinded reviewers judged each cell as scorable for both aneuploidy and pathogenic CNVs, for both validation sample series, according to the gestational age at sample collection. A maximum of five cells were sequenced, except that for twins all cells were sequenced.

The NGS data for all samples were scored by two ABMGG certified lab directors as pass or fail for aneuploidy and for smaller copy-number variants of ≥1 Mb. Cells that were scorable for both aneuploidy and ≥1 Mb deletions and/or duplications were considered high-quality cells. For the first study, 1.62 high-quality cells/sample were obtained; this numberincreased to 2.17 high-quality cells/sample for the second sample series. All scoring was blinded, but reviewers did know which cells were from the same pregnancy. Additional information they were provided included which pregnancies involved twins, the fetal sex as predicted by the cfDNA Y-PCR assay carried out as part of the SCT test, and the ultrasound-determined sex when available. The scores of the two reviewers were 89.0% concordant for pass/fail scores for each cell. The reviewers also scored every passing cell as normal (possibly including benign CNVs), aneuploid, or pathogenic CNV, and there was 100% concordance on these scores. All CNVs observed were previously well characterized with no rare CNVs of uncertain significance found. A common variant of uncertain significance (VUS) of deletion 15q11 (BP1-BP2) was observed in one instance. Out of all 95 samples included in the two studies, 52.6% had at least two high-quality cells that were scorable for both aneuploidy and smaller CNVs. As shown in Table 2 and Figure 3, out of all 32 singleton samples that were collected before 15 weeks of gestational age (because those have a better trophoblast yield), 56.3% had at least two high-quality cells for study 1, whereas for study 2 63.2% (38 pregnancies total) had at least two high-quality cells; the difference between the studies was primarily related to collection of a larger volume of blood in study 2. In 28.1% and 21.1% of samples in study 1 and study 2, respectively, only one high-quality cell was found. For 9.4% and 5.3%, only lower-quality data adequate for aneuploidy calling but not for smaller CNV detection were obtained, and for 6.3% and 10.5% of pregnancies no usable data at all were available. Similar data for pregnancies after 15 weeks of gestation showed that the success rate was much lower (Table S3).

**Table 2:** NGS Results for All Singleton Pregnancies at <15 Weeks of Gestational Age

**NGS Result**	**Study 1**	**Study 2**
Total pregnancies	32	38
≥2 high-quality cells	17 pregnancies (53.1%)	24 pregnancies (63.2%)
1 high-quality cell	10 pregnancies (31.3%)	8 pregnancies (21.1%)
Aneuploidy data only	3 pregnancies (9.4%)	2 pregnancies (5.3%)
No usable data	2 pregnancies (6.3%)	4 pregnancies (10.5%)

### Correlation with Normal and Abnormal Diagnostic Results Obtained from Amniocentesis or CVS

After completion of the SCT analysis, including a blinded final clinical interpretation, we reviewed the available clinical data and CVS and/or amniocentesis results for confirmation. All samples were given overall scores as normal, aneuploid, pathogenic CNV, or failed, and the overall score that each reviewer gave for all samples in which there was at least one high-quality cell was 100% concordant with the clinical data. There were also no examples of discordant findings in cells from same-sex twins. For study 1, 19 out of 42 pregnant women also underwent CVS and/or amniocentesis, whereas for study 2 this was 25 out of 53. For 33 out of these total 44 pregnancies, with complete data for both SCT testing and either amniocentesis or CVS (or both), and no evidence of mosaicism, there was complete concordance (27 normal and 6 aneuploidy; Tables 3 ). For eight cases, the CVS and/or amniocentesis results were normal, and the SCT testing failed (either there were no cells available for sequencing or sequenced cells did not yield any usable data). As discussed further below, the three remaining pregnancies had SCT findings that we interpret as confined placental mosaicism (CPM), whereas CVS and/or amniocentesis results were normal (Tables 3 and 4). If one focuses on the 33 pregnancies where adequate data from both SCT testing and either amniocentesis or CVS were available, both the sensitivity and the specificity were 100%. This excludes eight samples where SCT failed, and three that we interpret as likely to be CPM. For the 44 cases with CVS and/or amniocentesis data, the SCT failure rate was 18.1% (8/44). At least two cells were analyzed for 23 of those 44 pregnancies (52.3%); each cell from a particular pregnancy showed the same result. For nine cases (eight normal pregnancies and the trisomy18 pregnancy), there was only one cell available for analysis.

**Table 3:** Forty-Four Pregnancies with Available CVS or Amniocentesis Data

**SCT Testing**	**CVS and/or Amniocentesis Analysis**
27 normal results	27 normal results
6 aneuploid results∗	6 aneuploid results
8 failed analyses	8 normal results
3 mosaicism	3 normal results

∗ Included in the aneuploidy results were four trisomy-21 pregnancies (NIPT1303, NIPT1322, NIPT1336, and NIPT1443), one trisomy-18 pregnancy (NIPT1298), and one 47,XXY pregnancy (NIPT1501).

**Table 4:** Pregnancies Interpreted as Having Confined Placental Mosaicism

**Sample**	**GA**	**U/S**	***cf*NIPT**	**CVS and/or Amnio**	**SCT Testing**
NIPT1309	12 weeks, 1 day	normal	T13	direct & culture CVS normal (karyotype & CMA)	2/2 cells trisomy 13
NIPT1449	13 weeks, 3 days and 14 weeks, 2 days	normal	monosomy X	direct & culture CVS normal (karyotype & FISH)	4 cells 46,XX & 3 cells 45,X
NIPT1489	20 weeks, 2 days	abnormal	normal	amnio normal (karyotype & CMA)	1/1 cell trisomy 15

**Figure 4:**
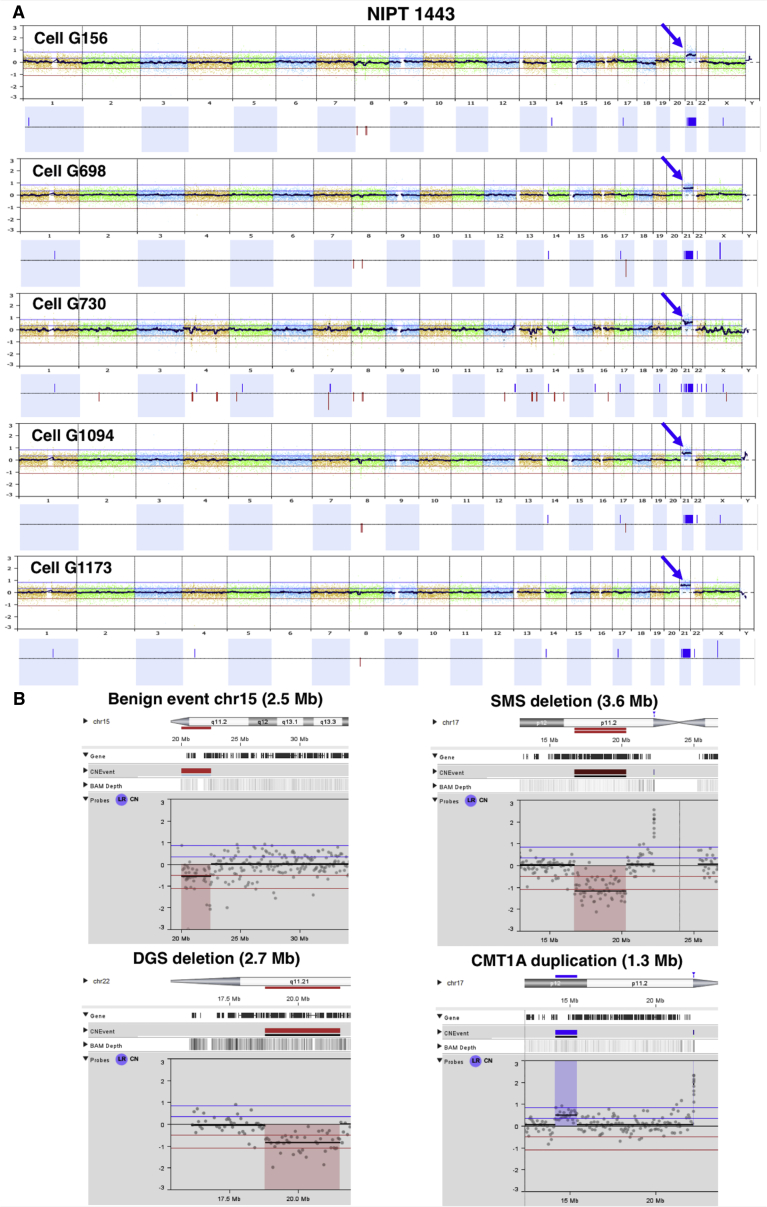
Examples of Aneuploidy and Subchromosomal CNV Detection (A) The NGS result of five single trophoblasts for a pregnancy in which the fetus was affected with trisomy 21. The clinical diagnosis was made by karyotype and chromosomal microarray after CVS. These plots were generated by comparison to a normal female reference cell. Cell G730 was judged to be in S phase and not scorable for smaller CNVs, although the trisomy is obvious despite the noise. The first example in (B) shows a detailed view of a trophoblast from a pregnancy in study 2. Chromosome 15 has a 2.5 Mb centromeric deletion, which is a benign, recurrent polymorphism, as frequently seen in our NGS data. The other three plots show the NGS result for a single lymphoblast carrying a 3.6 Mb Smith-Magenis deletion (SMS), a 2.7 Mb DiGeorge deletion (DGS), and a 1.3 Mb Charcot-Marie-Tooth duplication (CMT1A).

Multiple fetal aneuploidies confirmed by diagnostic testing were part of these sample series: four instances of trisomy 21, one instance of trisomy 18, and one fetus with a 47,XXY karyotype. One trisomy 21 example is shown in Figure 4A, illustrating the whole-genome plots of five different trophoblasts obtained for this case. Of note, the third cell in this example showed a pattern indicative of S-phase as described earlier, but it still clearly shows the trisomy.

### Confined Placental Mosaicism

As stated above, we found three instances of likely confined placental mosaicism (CPM) over the course of these two studies. For subject NIPT1309, the cell-free DNA-based screening test was positive for trisomy 13, whereas the follow-up CVS (both direct and cultured cells) showed a normal 46,XY profile by karyotype and chromosomal microarray (CMA). Ultrasound examination did not show any fetal abnormalities. The two trophoblast cells that were isolated from the SCT sample both showed a trisomy 13 profile. In a second pregnancy, NIPT1449 (Figure S2A), the cell-free DNA-based test had indicated “an increased risk for monosomy X.” Subsequent chromosome analysis on cultured CVS and FISH on direct CVS showed a normal 46,XX complement, and no follow-up amniocentesis was performed. No abnormal findings on ultrasound examination were reported. The seven scorable trophoblasts isolated from two different SCT blood samples indicated a mosaic result: four showed a normal 46,XX complement, whereas the other three cells showed a 45,X profile. It is of interest to note that an unusual number of poor-quality cells were found for this pregnancy: of those seven scorable cells, only four were of high quality, and another three were only scorable for aneuploidies. The remaining six cells were not scorable. For another sample (NIPT1489; Figure S2B), an unequivocal trisomy 15 was seen in the only trophoblast doublet obtained. The cell-free DNA-based testing showed a normal result, as did the amniocentesis analysis (direct and culture; karyotype and CMA were performed). Several fetal anomalies, including clubfoot, horseshoe kidney, and hydrocephalus, were noted on ultrasound examination.

### SCT Testing: CNV Resolution

There were no pregnancies involving a clinically diagnosed pathogenic CNV in these studies. Several benign CNVs frequently seen in polymorphic and repetitive regions around centromeres were reproducibly detected with our method (Figure 4B top left). For purposes of quality assurance and calibration for detection of deletions and duplications, we spiked into non-pregnant blood small numbers of lymphoblasts with known deletions or duplications. For lymphoblasts carrying a 3.6 Mb Smith-Magenis syndrome deletion, the deletion was detected in 100% of 26 cells with high-quality data (Figure 4B, top right). For DiGeorge syndrome lymphoblasts with a 2.7 Mb deletion, the deletion was detected in all nine high-quality cells (Figure 4B, lower left). For single lymphoblasts harboring the 1.3 Mb Charcot-Marie-Tooth type 1A duplication, the duplication was detected in 75% of 16 cells of high quality (Figure 4B, lower right).

## Discussion

The isolation and analysis of single circulating fetal or trophoblast cells could revolutionize the field of prenatal diagnosis because these cells offer an optimal source of fetal DNA. Although they are considered rare cells, multiple proof-of-principle publications have shown the value and feasibility of cell-based NIPT.^6^, ^7^, ^8^ In performing two SCT-testing validation studies, we report a yield of identified trophoblasts as 1.62 fully scorable cells/sample for the first sample series and 2.43 high-quality cells/sample for the second study. For these two studies, the downward trend in trophoblast recovery with increasing gestational age was found to be statistically significant. The increase in trophoblasts at an earlier gestational age could be explained by the process of trophoblast invasion’s leading to necessary uterine vascular remodeling during pregnancy.^11^ These invading trophoblasts enter the maternal veins and might in this manner end up in the maternal circulation.^12^ The trophoblast invasion process starts around 6–8 weeks of gestation and is generally completed by 19 or 20 weeks of gestation,^11^ hence possibly leading to a decreased trophoblast yield at a later gestational age. No significant correlation between maternal age and trophoblast yield was seen, and maternal BMI did not affect the yield significantly; in contrast, a stronger negative correlation with BMI was seen with cell-free DNA-based testing,^3^ although a slight downward trend in the number of trophoblasts was noted with increasing BMI. Another study also found a very weak trend toward lower cell numbers as BMI increased.^13^ Those authors concluded that “cell-based NIPT should not be hampered by an increased BMI.” The much-reduced effect of BMI on test performance is an important potential advantage of SCT testing over cell-free NIPT.

Focusing on the analysis of single trophoblast cells, we can learn several lessons from these studies. First, as we have shown before,^9^ it is critical to analyze each trophoblast individually because the quality of single cells can vary substantially. Some cells are in an apoptotic state involving genome-wide degradation and loss of chromosome arms or whole chromosomes. Apoptosis of trophoblasts in the maternal circulation is well described,^14^ and there was little way to anticipate what the frequency might be. Pooling apoptotic cells with high-quality trophoblasts prior to amplification would severely impact the overall result.^9^ Additionally, a few percent of cells are found to be in S phase, which also results in a disturbed whole-genome pattern because some parts of the genome will already have replicated before other parts, and the non-replicated regions can be misinterpreted as deletions. This phenomenon has been described earlier by Dimitriadou et al.,^15^ who concluded that a cell’s being in S phase can interfere with accurate CNV calling for the purpose of preimplantation genetic diagnosis. They illustrated that duplications from 9 to 25 Mb and deletions of 8 Mb were potentially missed in this scenario. Our results agree that these cells are inadequate for reliable CNV calling by NGS, but we found that S-phase cells are often still scorable for aneuploidy calling, as was seen in several of our trisomy cases (Figure S1). The preliminary validation studies performed here demonstrate that it is feasible to obtain multiple trophoblasts scorable for both aneuploidy and CNVs in more than half of all pregnancies tested. In the second study, which involved drawing 40 mL of maternal blood, two or more scorable cells were recovered in 57% of all pregnancies. Of note, for singleton pregnancies for which more than five cells were obtained, we did not perform NGS analysis on more than five cells. A central question is what number of scorable cells might represent an adequate result for clinical interpretation and a lab report. Although we began with a goal of three to five scorable cells per sample, it became clear that multiple pregnancies yielded only one, two, or occasionally zero high-quality cells—this remains an important limitation of our test. However, based on the very high concordance of multiple cells from individual singleton pregnancies, we become relatively confident of the accuracy of the clinical interpretation when two cells are found scorable by two independent, blinded, board-certified reviewers with full agreement on the conclusions. This does not rule out mosaicism.

Second, adequate genotyping of each trophoblast is essential. Despite our extensive experience and the application of strict criteria for trophoblast candidate selection by microscopy, occasionally a putative trophoblast cell was found to be of maternal origin. In the case of a male fetus, the confirmation is straightforward because an XY profile is clear from the whole genome plot obtained for CNV analysis, and 94% of all analyzed cells from a known male fetus were confirmed to be male. For female fetuses, single trophoblast genotyping needs to be built into the workflow so that that all analyzed cells are guaranteed to be of fetal origin. Of note, the probability that putative trophoblasts are of true fetal origin is higher when the cells appear in clusters, and we usually did not see these clusters for maternal cells.

Third, the results of analysis of multiple cells from the same pregnancy are remarkably consistent unless there is evidence of placental mosaicism or a multiple pregnancy. The ability of SCT testing to detect various forms of mosaicism is an important consideration. There are three types of CPM (types I, II, and III) and three types of true fetal mosaicism (TFM) (types IV, V, and VI);^16^ types I and II are the most common forms of CPM overall. CPM is not always benign; in a recent study on types II and III, the latter was found to be significantly associated with preterm births, newborns who were small for their gestational age, and adverse pregnancy outcomes.^17^ Both types II and V involve mesenchymal cells only and would thus not be detected by our SCT assay, which only examines cytotrophoblastic cells. In our 95-sample series, we encountered three instances of likely CPM, and these were most compatible with type I CPM. In two of those pregnancies there was independent evidence for the presence of CPM from other clinical results. For the third pregnancy, we interpret the recovery of the only cell showing trisomy 15 from a pregnancy with a normal cell-free NIPT and amniocentesis outcome as most likely originating from low-level CPM. Although mosaicism overall is detected in about 2% of CVS analyses and 1% for CPM type I specifically, the gestational age and the number of cells studied can affect detection rates. Hence, in this situation, pursuing a confirmatory CVS or amniocentesis would be recommended. The time frame for SCT testing, however, is of great advantage because it can be done early enough in the pregnancy to allow ample time for follow-up testing. Several samples collected at 10 weeks of gestation or earlier were included in this study and produced satisfactory results, and we are currently collecting more samples as early as 6 or 7 weeks of gestational age. It is also important to note that other factors, such as ultrasound examination, will help in evaluating a SCT test result.

Fourth, the analysis of multiple pregnancies can prove a challenging task, but SCT testing has advantages over cell-free DNA-based NIPT because each fetus can be tested separately. Although, in general, a higher number of trophoblasts is obtained for multiples, this does not guarantee recovery of cells from all fetuses. If twins are of opposite sexes, it is straightforward to determine which cells are from which fetus. Although for three out of the five opposite-sex twin pregnancies described here cells from both twins were analyzed, for the two other pregnancies data were available for only one fetus, indicating that more method improvements for twin analysis are necessary. Our current method is also limited in the sense that the zygosity of the multiples is not determined and that it is not always possible to track from which fetus a certain trophoblast originated (e.g., when both twins are of the same sex and/or when no obvious ultrasound findings can give any indication). Nevertheless, SCT testing can still offer valuable information on twin and multiple pregnancies because each trophoblast is analyzed and evaluated separately. A DNA sample from the father is not needed for SCT testing unless there is a question of whether a finding in the fetus is *de novo* or inherited.

Finally, our method allows reliable detection of CNVs down to 1 Mb in size, as we illustrated previously,^9^ and is here confirmed again by the detection of small, benign, recurrent CNVs (Figure 4). Although these pericentromeric repetitive sequences are often excluded in microarrays, these regions were included in the NGS analysis if reads could be uniquely mapped in the genome. We have also shown that the use of spiked-in single lymphoblast cells can be very helpful for quality assurance regarding detection of CNVs of various sizes.

It is difficult to compare these results to the National Institute of Child Health and Human Development Fetal Cell Isolation Study (NIFTY) study from 17 years ago.^18^ That study focused on fetal nucleated red blood cells and fluorescence *in situ* hybridization (FISH) detection of aneuploidy, whereas this study focuses on trophoblasts and detection of CNVs down to 1–2 Mb. Putting aside these major differences, the NIFTY study found “at least one aneuploid cell in 74.4% of cases of fetal aneuploidy,” whereas this study found at least one aneuploid cell in 100% of affected fetuses.

The results from SCT testing are all-or-none conclusions, such as whether a particular aneuploidy or pathogenic CNV is present or absent in the cell being analyzed. This is similar to cytogenetic chromosomal microarray data and can thus be considered a qualitative result, more characteristic of a diagnostic test. In contrast, cell-free NIPT can only provide a probability that a particular aneuploidy or pathogenic CNV is present or absent, and this limited ability is more characteristic of a screening test.

We have mentioned a number of limitations, including the inability to obtain high-quality data for multiple cells from every fetus. Some cells are apoptotic or in S phase, but because all cells are analyzed individually, these cells do not interfere with the interpretation of high-quality cells. Although the request was to draw blood prior to CVS or amniocentesis, this was not always achieved in the busy clinic environment. We did not find a significant difference in cell recovery when blood was drawn either before or after CVS (in 12 cases prior to, in 16 after CVS) or amniocentesis (12 prior to, four after), but the number of samples is low and too small to allow comparison of the effect of length of time between the procedure and blood draw. The detection of CPM can bring both some advantages and some disadvantages. Detecting mosaicism in general is an advantage because it provides information about the fetus, such as providing the opportunity to detect uniparental disomy or true fetal mosaicism and could easily be followed up with CVS and/or amniocentesis. Our method differs from CVS in that it fails to detect mesenchymal CPM. Although the current higher costs and limited throughput may be disadvantages initially, we believe that these limitations can be solved through (technical) improvements and automation.

Is the test clinically useful in its present form? Opinions are likely to differ. We estimate that the cost of testing with the current protocol would be at least $3,000, and the throughput would be a constraint. We expect that improvements could lower costs and increase throughput substantially. The turnaround time would be 2–3 days longer than that for cell-free NIPT. In light of the 15.8% no-result rate for CNVs and the 10.5% no-result rate for aneuploidy in study 2 as shown in Table 2, there is clear need for improvement. Any test failures could be followed up through CVS or amniocentesis. Although the recovery of two or more high-quality cells from >95% of fetuses would make the test more ready for clinical use, even in its current form it could be an attractive clinical option for early testing of pregnancies at high risk.

In conclusion, SCT analysis is potentially a powerful tool for prenatal testing and diagnosis. We are optimistic that the recovery of trophoblasts can be improved. SCT testing has the potential to deliver a diagnostic result instead of being merely a screening test if an adequate number of trophoblast cells can be obtained for every sampled pregnancy. A longer-term goal would be to detect all *de novo* point mutations in a fetus.

## Declaration of Interests

Baylor Genetics (BG) is a diagnostic laboratory partially owned by Baylor College of Medicine. Several authors are located at BG, as indicated, and A.L.B. and I.B.V. have consulting or committee roles at BG. A.L.B. is a founder of Luna Genetics.

## Web Resources

OMIM, https://www.omim.org/

## Supplemental Data

Document S1. Figures S1, S2 and Tables S2, S3

Table S1. Overview of Sample Characteristics for Samples from Both Studies

Document S2. Article plus Supplemental Data
