# Supplementary material for: Validation Studies for Single Circulating Trophoblast Genetic Testing as a Form of Noninvasive Prenatal Diagnosis
Source: Am J Hum Genet. 2019 Nov 27;105(6):1262–73. doi: 10.1016/j.ajhg.2019.11.004 (PMC6904821; doi:10.1016/j.ajhg.2019.11.004)
Supplement: Document S1. Figures S1, S2 and Tables S2, S3 [file mmc1.pdf]

The American Journal of Human Genetics, Volume *105*

Supplemental Data

Validation Studies for Single Circulating Trophoblast Genetic Testing as a Form of Noninvasive Prenatal Diagnosis

Liesbeth Vossaert, Qun Wang, Roseen Salman, Anne K. McCombs, Vipulkumar Patel, Chunjing Qu, Michael A. Mancini, Dean P. Edwards, Anna Malovannaya, Pengfei Liu, Chad A. Shaw, Brynn Levy, Ronald J. Wapner, Weimin Bi, Amy M. Breman, Ignatia B. Van den Veyver, and Arthur L. Beaudet

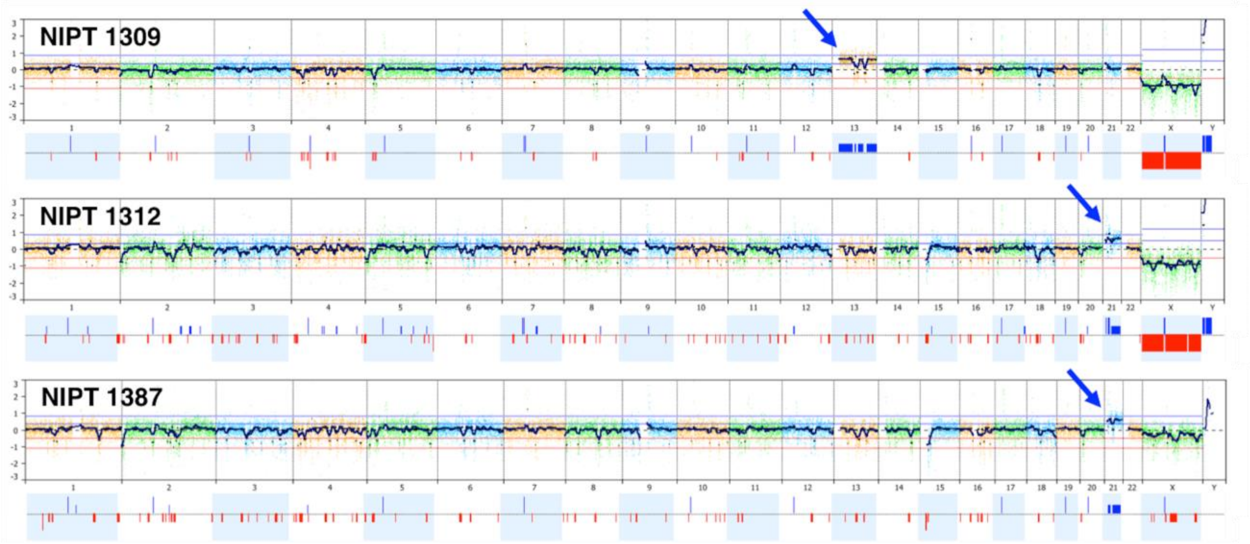

Figure S1. S-phase cells for aneuploidy calling. This figure includes three examples of single trophoblasts each showing a pattern indicating the cell being in S-phase, but that are nevertheless still useful for calling a trisomic event. These whole genome plots were generated by comparing to a normal female reference: the two top cells were obtained from a male fetus, while the third cell was recovered from a female pregnancy.

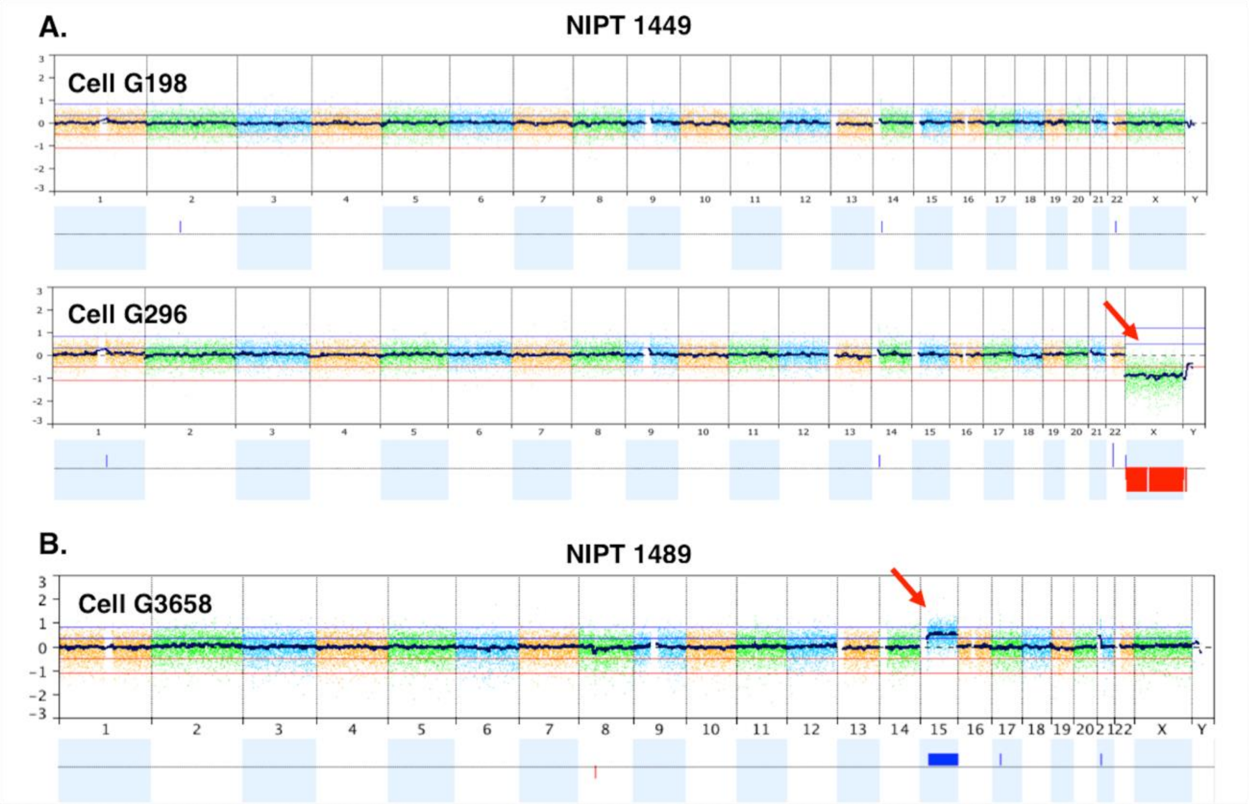

Figure S2. Confined placental mosaicism. The two plots in panel A show two of the seven scorable trophoblasts found for case NIPT1449, illustrating the mosaic 45,X/46,XX result that was seen for this sample. Cell G198 shows a normal 46,XX complement compared to a normal female reference, while cell G296 shows a clear loss of one copy of X. Panel B shows the trophoblast doublet that was analyzed for NIPT1489, in which a clear trisomy 15 was seen.

Table S2. Overview of samples from twin pregnancies

Case	GA	Fetal sex	Cells/mL	Total # of cells sequenced	# of high-quality cells	# of cells with aneuploidy data only	
**NIPT1329**	12w3d	M + F	0.33	7	4	3	Cells from both fetuses
**NIPT1334**	13w3d	M + M	0.40	6	4	0	*(+2 poor quality cells)*
**NIPT1432**	19w	M + F	0.11	1	1	0	Data for only 1 fetus
**NIPT1441**	13w6d	M + F	0.77	10	3	7	Data for only 1 fetus (only female cells were recovered)
**NIPT1452**	13w2d	F + F	0.65	8	2	2	*(+4 poor quality cell)*
**NIPT1482**	13w	M + F	0.25	5	3	1	*(+1 poor quality cell)*<br>Cells from both fetuses
**NIPT1495**	12w	M + M	0.33	9	5	1	*(+1 poor quality cell; +2 cells of maternal origin)*
**NIPT1496**	12w2d	M + F	0.40	10	7	2	*(+1 poor quality cell)*<br>Cells from both fetuses

Table S3. NGS results for all singleton cases collected ≥15 weeks gestation

NGS result	Study 1 (8 cases)	Study 2 (9 cases)
≥ 2 high-quality cells	1 case (12.5%)	1 case (11.1%)
1 high-quality cell	3 cases (37.5%)	6 cases (66.7%)
Aneuploidy data only	2 cases (25.0%)	1 case (11.1%)
No usable data	2 cases (25.0%)	1 case (11.1%)
